# P31 The effects of topical 5-fluorouracil treatment of *in situ* squamous cell carcinomas on antimicrobial resistance and small colony variant formation in *Staphylococcus aureus*

**DOI:** 10.1093/jacamr/dlaf230.038

**Published:** 2025-12-04

**Authors:** Daniella Massri, Zoe C Venables, Benjamin Evans

**Affiliations:** Norfolk and Norwich University Hospital, Norwich, UK; Norfolk and Norwich University Hospital, Norwich, UK; University of East Anglia, Norwich, UK; University of East Anglia, Norwich, UK

## Abstract

**Background:**

Squamous cell carcinoma (SCC) is a prevalent skin malignancy. Its precursor lesion, in-situ squamous cell carcinoma (iSCC), is often treated with topical 5-fluorouracil (5-FU). 5-FU may be linked with antibiotic resistance in *Staphylococcus aureus*, a common skin commensal with pathogenic potential. However, the effects of 5-FU on antibiotic resistance and pathogenicity in *S. aureus* remain unclear.

**Objectives:**

To investigate the effects of Topical 5-FU exposure in iSCC on *S. aureus*, with a focus on the formation and characteristics of Small Colony Variant (SCVs) and implications for antibiotic resistance, pathogenicity, biofilm formation and infection risk.

**Methods:**

Antibiotic susceptibility and biofilm formation were tested in *S. aureus* NCTC 6571 SCVs and normal colony variants (NCVs). A 21 day evolution experiment, followed by biofilm formation and antibiotic sensitivity testing, as well as a protease assay were performed on *S. aureus* NCTC 6571, F77 and ATCC 25923 isolates. Clinical sampling to collect *S. aureus* from iSCC lesions was then conducted.

**Results:**

Minor differences in antibiotic susceptibility were observed among *S. aureus* NCTC 6571 SCVs and normal colony variants (NCVs). The MIC of linezolid was reduced against SCVs compared to NCVs. Biofilm formation was increased in some SCVs and decreased in the ancestor and NCVs. *S. aureus* NCTC 6571, ATCC 25923 and F77 biofilms were then exposed to 5-FU for 21 days. Long-term 5-FU exposure led to increased SCVs in *S. aureus* F77. Biofilm formation was varied, with 5-FU associated reductions in NCTC 6571 and ATCC 25923, while F77 displayed minimal changes. Protease activity in NCTC 6571 was reduced following long-term exposure. 43% of clinical AK isolates displayed *S. aureus* colonization, but no SCVs.

**Conclusions:**

Our study contributes to a comprehensive understanding of the effects of 5-FU on *S. aureus* skin infections, particularly regarding the wider implications for iSCC and SCC treatment and antimicrobial resistance. Isolates from this study require further characterization, including identification of phenotypic characteristics, and antibiotic susceptibility testing. The observed strain-specific responses to 5-FU emphasize the need for continued research involving various strains. The emergence of SCVs may have clinical consequences, particularly contributing to infection persistence, resistance to treatment and increased risk of infection and recurrence in patients with SCC. Further studies are also needed to clarify the long-term implications.

